# CMV pp65 and IE-1 T cell epitopes recognized by healthy subjects

**DOI:** 10.1186/1479-5876-5-17

**Published:** 2007-03-28

**Authors:** Stefanie L Slezak, Maria Bettinotti, Silvia Selleri, Sharon Adams, Francesco M Marincola, David F Stroncek

**Affiliations:** 1 Department of Transfusion Medicine, Warren G. Magnuson Clinical Center National Institutes of Health, Bethesda, Maryland, USA

## Abstract

**Background:**

Adoptive immune and vaccine therapies have been used to prevent cytomegalovirus (CMV) disease in recipients of hematopoietic progenitor cell transplants, but the nature of T cell responses to CMV have not been completely characterized.

**Methods:**

Peptide pools and individual peptides derived from the immune-dominant CMV proteins pp65 and IE-1 and antigen-specific, cytokine flow cytometry were used to characterize the prevalence and frequency of CD4+ and CD8+ memory T cells in 20 healthy CMV-seropositive subjects.

**Results:**

CD8+ T cell responses to pp65 were detected in 35% of subjects and to IE-1 in 40% of subjects. CD4+ T cell responses to pp65 were detected in 50% of subjects, but none were detected to IE-1. Several new IE-1 HLA class I epitopes were identified, including 4 restricted to HLA-C antigens. One region of IE-1 spanning amino acids 300 to 327 was rich in class I epitopes. The HLA class I restrictions of IE-1 peptides were more promiscuous than those of pp65 peptides.

**Conclusion:**

Since naturally occurring CD4+ and CD8+ T cell responses to pp65 were detectable in many subjects, but only CD8+ T cell responses to IE-1 were detected, pp65 may be better than IE-1 for use in vaccine and adoptive immune therapies.

## Background

Cytomegalovirus (CMV) is a persistent virus in normal hosts in which primary infection is typically controlled through a combination of adaptive and innate immune responses. Viral latency follows primary infection and continues for life, usually without major symptoms. Although 60% to 80% of adults that test seropositive for CMV antibodies show no symptoms of infection, in immunocompromised hosts, such as patients undergoing hematopoietic stem cell transplantation (HSCT) or recipients of organ transplants, CMV can cause severe disease[1]. In the past 10 years, the incidence of CMV disease in allogeneic HSCT recipients during the first 100 days after transplantation fell from 40% to 5% as a result of improved diagnosis and prophylactic or preemptive treatment with antiviral drugs. However, the effect of the improved therapy has delayed disease onset to 6 to 12 months after transplantation, and the risk of severe CMV disease remains in 10% of allogeneic HSCT recipients[2].

In order to control rather than delay the incidence of CMV disease after HSCT, the host's anti-CMV immunity must be restored. Because CMV immunotherapy is growing in importance, the study of the immune response to CMV is clinically relevant. T cell immunity is believed to be the most important component in immune response to CMV disease. This is supported by the fact that Natural Killer cells recover early after HSCT, before the peak incidence of CMV disease, and by the failure of infusion of immunoglobulin (Ig) preparations containing antiviral antibodies to control the disease [3,4]. In contrast, clinical trials involving adoptive transfer of CMV-specific T cells were successful in restoring immunity against the virus [5,6]. An alternative to adoptive immunotherapy is vaccination of HSCT donors against CMV with the aim of providing specific immunity to the recipient for protection against primary infection or reactivation[7].

Two CMV proteins, phosphoprotein 65 (pp65) and immediate early protein-1 (IE-1), have been found to be major targets of the cellular immune response [8-12]. Immunodominant epitopes in these proteins have been defined for some human leukocyte antigen (HLA) alleles, but further definition for specific major-histocompatibility complex (MHC) classes and HLA allelic restrictions could be useful for adoptive immune therapy, vaccine therapy, and in screening for reactivation after immunosuppression[13,14].

The purpose of this study was to characterize the natural repertoire of immunodominant pp65 and IE-1 epitopes in 20 CMV-seropositive, healthy subjects. Donor CD4+ and CD8+ T cell responses to CMV pp65 and IE-1 15-mer peptide libraries were defined and followed downstream to the individual 15-mer and nanomer peptides, respectively. This process identified epitopes without biases introduced by focusing on specific immunodominant regions for individual HLA types.

## Methods

### Study design

**Figure 1:**
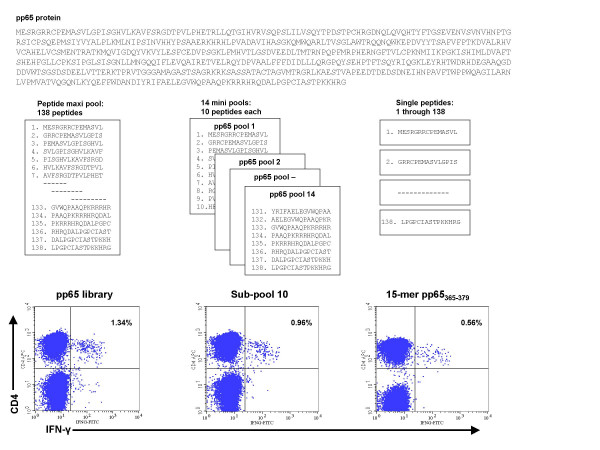
**Identification of CMV pp65 epitopes using 15-mer and nanomer peptides**. PBMCs were stimulated with a library of 138 15-mer peptides overlapping by 11 residues and covering the entire pp65 protein. IFN-γ flow cytometry was used to detect T cell responses. To identify specific epitopes, PBMCs were stimulated with subpools containing 10 overlapping 15-mer peptides. PBMCs from donors reactive with CD4+ T cells were tested further with individual 15-mers. The results of analysis of CD4+ T cell responses in donor 14 to pp65 peptides are shown.

**Figure 2:**
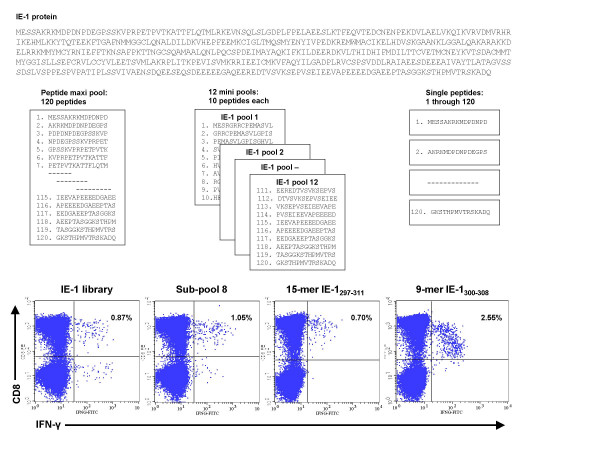
**Identification of CMV IE-1 epitopes using 15-mer and nanomer peptides**. PBMCs were stimulated with a library of 120 15-mer peptides overlapping by 11 residues and covering the entire IE-1 protein. IFN-γ flow cytometry was used to detect T cell responses. To identify specific epitopes, PBMCs were stimulated with peptide subpools containing 10 overlapping 15-mer peptides. PBMCs from donors with reactive CD8+ T cells were tested further with individual 15-mers and nanomers. The results of analysis of CD8+ T cell responses in donor 6 to IE-1 peptides are shown.

In this study, 20 CMV-seropositive and 5 CMV-seronegative healthy donors were evaluated. To determine the CD8+ and CD4+ T cell responses to CMV in the 25 donors, peripheral blood mononuclear cells (PBMCs) were stimulated in vitro with a cocktail of 15-mer peptides overlapping by 11 residues and covering the pp65 and IE-1 proteins. Antigen-specific cytokine flow cytometry was used to detect specific T cell responses[15,16]. To define immunodominant epitopes, PBMCs were also stimulated with subpools of pp65 and IE-1, each subpool containing 10 overlapping 15-mer peptides. PBMCs reactive to a subpool were then tested further against the individual peptides contained within the subpool to determine the reactive 15-mer(s). In order to further characterize epitopes presented to CD8+ T cells by HLA class I antigens, PBMCs from donors with reactive CD8+ cells were tested again with nanomer peptides spanning the reactive 15-mer peptide and overlapping at 8 amino acids (Figures 1 and 2). These studies were approved by a National Institutes of Health Institutional Review Board on the use of Human Subjects in Research.

### Collection of PBMCs

PBMC concentrates from 20 CMV-seropositive and 5 CMV-seronegative healthy donors were collected by apheresis, (Fenwal CS-3000 Plus, Baxter Healthcare Corp, Deerfield, IL) and peripheral blood mononuclear cells were isolated by ficoll-hypaque density gradient separation and frozen in aliquots of 1 × 10^8 ^cells. Donors were typed for HLA class I and class II alleles using sequence-specific primers and, in some cases, direct sequencing (HLA Laboratory, DTM, CC, NIH, Bethesda, MD). The presence of IgG and IgM CMV antibodies in each subject was analyzed by passive agglutination (BD, Microbiological Systems, Cockeysville, MD).

### Peptide libraries and pools

Libraries for CMV proteins pp65 and IE-1 were made up of peptides 15 amino acids long that overlapped by 11 residues and covering the complete pp65 (CMV Towne) [17] and IE-1 (CMV AD169) proteins[18]. The pp65 library was made up of 138 peptides and the IE-1 library of 120 peptides, and both were commercially synthesized (Princeton Biomolecules, Langhorne, PA). Peptides were diluted in dimethyl sulfoxide and pooled into cocktails containing the complete pp65 and IE-1 protein sequences and into subpools containing 10 consecutive peptides each. Pp65 was divided into 13 subpools of 10 peptides and 1 subpool of 8 peptides, and IE-1 was divided into 12 subpools of 10 peptides (Figures 1 and 2).

### PBMC stimulation and flow cytometric assessment

T cell activation was assessed by measuring intracellular interferon gamma (IFN-γ) production by flow cytometry after PBMC stimulation. PBMCs were plated at 1–5 × 10^6 ^per mL in 24-well plates with 1 mL of complete medium (CM) supplemented with 10% heat-inactivated human AB serum, 10 μM HEPES buffer, 100 U/mL penicillin-streptomycin, 0.03% L-glutamine, and 10 mg/mL ciprofloxacin. Following overnight resting at 37°C, PBMCs were stimulated with 1 μg/mL of pp65 and IE-1 libraries, sub-pools, or individual peptides and 0.5 mg/mL of staphylococcal enterotoxin B (SEB) (Sigma, St Louis, MO) as a positive control.

After 1 h of incubation at 37°C, 2 μg/ml of Brefeldin A (BFA) (Sigma) was added, and the cells were incubated for an additional 5 h before harvesting, fixed in FACS Lysing Solution (BD Biosciences, San Jose, CA) at room temperature and rested overnight at 4°C. The following day PBMCs were washed, permeabilized in Perm2 Solution and stained with monoclonal antibodies against CD3-PerCP, CD8-PE, CD4-APC and IFNγ-FITC (all from BD Biosciences) and incubated at 4°C for 30 minutes. Mouse Ig isotype controls were also used (BD Biosciences). The 2-laser FACSCalibur flow cytometer and CellQuest Pro software (BD Biosciences) were used for analysis by acquiring 200,000 events, and determining the viable lymphocyte population by light scatter. Gates were designed for CD3+ expression and for characterization of CD8+ and CD4+ subpopulations. The proportion of reactive CD8+ and CD4+ cells was expressed as a percent of the total number of CD8+ and CD4+ cells analyzed, respectively.

Reactive populations met 2 criteria: (1) well-defined cell population reactive with both IFN-γ and CD8+ or CD4+, and (2) the percentage of IFN-γ producing cells was greater than 3 standard deviations of the percentage of unstimulated IFN-γ producing cells. Scattered reactive cells were not sufficient to be considered a positive population.

### Determination of HLA restriction

Epitope HLA restrictions were determined by testing the ability of peptide-pulsed Epstein-Barr Virus (EBV) transformed lymphoblastoid B cell lines (EBV-LCL) to stimulate T cells that were sensitized in vitro with the same peptide. In vitro sensitization involved an 8 to10 day cell culture in the presence of the determined CMV nanomers or 15-mers. PBMCs were plated at a concentration of 1 × 10^7 ^PBMC per mL in a 24 well-plate with 2 mL CM, and directly stimulated with 1 μg/mL of pre-determined peptide. Recombinant human interleukin-2 (50 U/mL, Chiron, Emeryville, CA) was added to the cells at 24 hours and every other day.

Partially HLA matched EBV-LCL panels were developed for each donor, to match HLA class I or class II antigens. EBV-LCLs in CM were pulsed with 1 μg/mL of peptide and incubated at 37°C for 1 h, mixing vigorously at 30 m intervals. Washed EBV-LCLs were resuspended in CM at 1 × 10^6 ^cells per mL, and 1 mL was added to the sensitized cells which were plated according to the methods described above. The plates were centrifuged for 2 minutes at 500 rpm and incubated for 1 h at 37°C before adding 2 μg/mL of BFA and incubating for an additional 5 h. Samples were treated and analyzed as described above.

## Results

Peripheral blood was obtained from 25 healthy subjects; 20 CMV-seropositive and 5 CMV-seronegative, similar in age, gender, and race (Table 1). The HLA types of the subjects were representative of the general population (Table 2). PBMCs from all 25 subjects were tested against the complete pp65 peptide library and subpools (Figure 1) and the complete IE-1 peptide library and subpools (Figure 2).

**Table 1:** Age, Gender, and Race of Subjects Studied

	**CMV-Seropositive n = 20**	**CMV-Seronegative n = 5**	**All n = 25**
**Age, years**			

Median	45.1	42.8	44.6
Range	17–64	35–49	17–64
**Gender**			

Male, %	80	80	80
**Race**			

White, %	55	60	56
Black, %	40	20	36
Hispanic, %	5	0	4
Unknown, %	0	20	4

**Table 2:** HLA Genotype of the 20 CMV-Seropositive Subjects Studied

		**HLA Type**					
		
**Donor**	**Race**	**Class I**			**Class ll**		
		A*	B*	C*	DRB1*	**DRB-**	DQB1*

1	Black	01, 0301	15, 81	05, 18	0901, 11	3*00, 4*01	02, 06
2	Caucasian	24, 32	07, 40	02, 07	0401, 1302	3*0301, 4*0103	03, 0609
3	Caucasian	01, 31	07, 27	0202, 0702	0801, 1501	5*01, 5*01	04, 06
4	Black	30, 3601	53, 53	04, 04	03, 1001	3*01, 3*01	04, 05
5	Hispanic	11, 68	44, 45	05, 06	0404, 1101	3*02, 4*0103	03, 03
6	Black	26, 30	35, 57	04, 18	11, 16	3*02, 5*02	0301, 05
7	Caucasian	01, 0201	07, 35	04, 07	0401, 0701	4*01, 4*01	0303, 03
8	Black	0202, 30	4901, 53	04, 07	13031, 1503	3*02, 3*02	02, 06
9	Caucasian	23, 32	35, 41	04, 17	11, 11	3*02, 3*02	03, 06
10	Black	0301, 68	53, 58	04, 06	0701, 1302	3*0301, 4*0401	0202, 0604
11	Black	26, 74	15, 5802	0602, 1601	0102, 1101	3*0202, 3*0202	0301, 0501
12	Black	0202, 68	5301, 5301	04, 04	03021, 1503	3*01, 5*01	04, 06
13	Caucasian	0201, 0201	15, 18	03, 07	13, 1501	3*02, 5*01	06, 06
14	Caucasian	010101, 2601	070201, 3801	070201, 120301	0402, 1101	3*02, 4*0103	0301, 0302
15	Caucasian	0201, 30	18, 44	0501, 0501	04, 13	3*02, 4*01	03, 06
16	Caucasian	0201, 11	07, 40	03, 07	0301, 1501	3*01, 5*01	02, 06
17	Caucasian	0201, 0201	15, 18	03, 07	13, 1501	3*02, 5*01	06, 06
18	Black	2301, 24	07, 18	0202, 0702	07, 15	4*01, 5*01	02, 06
19	Caucasian	03, 29	35, 44	04, 1601	01, 0701	4*01, 4*01	0202, 0501
20	Caucasian	03, 11	07, 5601	01, 07	0101, 0701	4*01, 4*01	02, 0501

### T cell responses to the complete pp65 and IE-1 peptide libraries

Neither the complete pp65 or IE-1 libraries stimulated CD8+ or CD4+ T cells from the 5 CMV-seronegative subjects (Table 3). The proportion of CD8+ cells producing intracellular IFN-γ in response to the pp65 library ranged from 0.02% to 0.09%, and in response to the IE-1 library ranged from 0.00% to 0.05%. The proportion of CD4+ cells producing IFN-γ in response to the pp65 library ranged from 0.00% to 0.04% and in response to the IE-1 library ranged from 0.01% to 0.04%. PBMCs were also tested against the subpools, none of which induced significant IFN-γ.

**Table 3:** Proportion of Peripheral Blood CD8+ and CD4+ T Cells from 5 Healthy CMV-Seronegative Subjects Producing Intracellular IFN-γ Following Stimulation With a Library of 138 CMV pp65 or 120 CMV IE-1 15-mer Peptides

		**Proportion of T Cells Producing Intracellular IFN-γ(%) in Each Donor**				
**Library**	**Cell Type**	**1**	**2**	**3**	**4**	**5**

pp65	CD8+	0.02	0.03	0.03	0.08	0.09
IE-1	CD8+	0.04	0.02	0.01	0.00	0.05

pp65	CD4+	0.00	0.03	0.02	0.02	0.03
IE-1	CD4+	0.01	0.01	0.02	0.01	0.04

Testing PBMCs from each of the 20 CMV-seropositive subjects revealed that the pp65 and IE-1 libraries stimulated CD8+ T cells in 7 (35%) and 8 (40%) subjects, respectively. IFN-γ producing cells ranged from 0.12% to 0.70% for pp65 and 0.32% to 2.06% for IE-1 (Table 4). CD4+ cells were stimulated by the pp65 library in 10 (50%) subjects with the percent of IFN-γ producing cells ranging from 0.11% to 3.33%, but were not stimulated in any donors by the IE-1 library (Table 4).

**Table 4:** Proportion of Peripheral Blood CD8+ and CD4+ T Cells From 20 Healthy CMV-Seropositive Subjects Producing Intracellular IFN-γ Following Stimulation With a Library of 138 CMV pp65 or 120 CMV IE-1 15-mer Peptides

		**Proportion of T Cells Producing Intracellular IFN-γ(%) in Each Donor**																			
		
**Library**	**Cell**	**1**	**2**	**3**	**4**	**5**	**6**	**7**	**8**	**9**	**10**	**11**	**12**	**13**	**14**	**15**	**16**	**17**	**18**	**19**	**20**
pp65	CD8+	0.02	0.06	***0.37***	0.10	***0.27***	0.13	***0.70***	0.13	0.13	***0.27***	0.11	0.18	***0.12***	***0.27***	0.07	0.08	0.06	0.05	***0.18***	0.03
IE-1	CD8+	0.05	***0.46***	***2.06***	0.04	***1.65***	***0.87***	***1.37***	0.13	0.15	***0.33***	***0.32***	0.06	0.02	0.12	***1.41***	0.05	0.05	0.02	0.03	0.08

pp65	CD4+	***0.16***	0.06	0.00	0.06	0.11	***0.76***	***0.11***	***0.65***	0.08	***0.21***	***3.33***	***0.18***	***0.20***	***1.34***	0.23	0.02	***0.15***	0.03	0.04	0.01
IE-1	CD4+	0.02	0.04	0.01	0.03	0.02	0.14	0.03	0.05	0.01	0.02	0.08	0.06	0.04	0.13	0.09	0.02	0.05	0.02	0.00	0.10

*Positive CD8+ T cell assays are indicated in bold, italics.

### CD8+ T cell responses to pp65 peptides

Testing of PBMCs from all 20 CMV-seropositive donors against the pp65 subpools revealed that all 7 subjects with CD8+ cells that were reactive to the pp65 library reacted to at least 1 pp65 subpool. We identified 5 reactive pp65 subpools, with subpool 11 stimulating CD8+ cells from 3 subjects (Table 5).

**Table 5:** Proportion of Peripheral Blood CD8+ T Cells From 20 Healthy CMV-Seropositive Subjects Producing Intracellular IFN-γ Following Stimulation With a Library of 138 CMV pp65 15-mer Peptides and Each of 14 subpools of 10 Peptides*

	**CD8+ T Cells Producing Intracellular IFN-γ in Each Donor (%)**																			
	**1**	**2**	**3**	**4**	**5**	**6**	**7**	**8**	**9**	**10**	**11**	**12**	**13**	**14**	**15**	**16**	**17**	**18**	**19**	**20**

**Library**	0.02	0.06	***0.37***	0.10	***0.27***	0.13	***0.70***	0.13	0.13	***0.27***	0.11	0.18	***0.12***	***0.27***	0.07	0.08	0.06	0.05	***0.18***	0.03
**Subpool**																				
**1**	0.01	0.04	0.07	0.03	0.01	0.02	0.02	0.02	0.01	0.01	0.12	0.09	0.14	0.03	0.01	0.08	0.01	0.00	0.02	0.03
**2**	0.01	0.05	0.02	0.03	0.17	0.05	0.11	0.05	0.06	0.03	0.03	0.08	0.01	0.03	0.06	0.08	0.03	0.03	0.03	0.02
**3**	0.02	0.02	0.07	0.13	0.04	0.06	0.15	0.01	0.03	0.02	0.03	0.16	0.03	0.04	0.03	0.03	0.05	0.01	***0.27***	0.02
**4**	0.02	0.04	0.03	0.01	0.10	0.08	0.18	0.08	0.06	0.09	0.01	0.08	0.02	0.05	0.07	0.02	0.00	0.05	0.05	0.02
**5**	0.04	0.01	0.02	0.03	***0.13***	0.04	0.08	0.07	0.11	0.00	0.04	0.06	0.08	0.02	0.03	0.08	0.01	0.01	0.03	0.02
**6**	0.00	0.01	0.00	0.14	0.08	0.03	0.04	0.05	0.08	***0.21***	0.05	0.06	0.03	0.03	0.11	0.08	0.03	0.04	0.01	0.02
**7**	0.03	0.06	0.03	0.03	0.04	0.01	0.11	0.07	0.07	0.08	0.03	0.07	0.08	0.15	0.03	0.03	0.02	0.10	0.03	0.02
**8**	0.02	0.00	0.03	0.01	0.03	0.10	0.01	0.10	0.03	0.01	0.03	0.08	0.03	0.02	0.02	0.10	0.02	0.04	0.03	0.05
**9**	0.05	0.03	0.00	0.06	0.02	0.03	0.03	0.02	0.03	0.02	0.05	0.12	0.02	0.04	0.02	0.05	0.03	0.04	0.03	0.13
**10**	0.06	0.02	0.00	0.05	0.02	0.10	0.14	0.06	0.03	0.04	0.06	0.05	0.02	0.08	0.03	0.06	0.03	0.03	0.04	0.02
**11**	0.02	0.02	***0.17***	0.01	0.02	0.06	***0.36***	0.04	0.06	0.04	0.05	0.12	0.01	***0.13***	0.01	0.05	0.01	0.01	0.00	0.02
**12**	0.12	0.08	0.03	0.00	0.07	0.02	0.06	0.05	0.09	0.01	0.03	0.04	0.01	0.03	0.00	0.08	0.02	0.03	0.02	0.05
**13**	0.05	0.05	0.02	0.00	0.01	0.02	0.06	0.06	0.07	0.04	0.08	0.07	***0.12***	0.03	0.04	0.03	0.09	0.03	0.01	0.02
**14**	0.03	0.01	0.02	0.01	0.10	0.04	0.06	0.07	0.08	0.08	0.06	0.07	0.02	0.01	0.03	0.02	0.02	0.05	0.04	0.00

* Subpool 14 contained 8 peptides

Positive CD8+ T cell assays are indicated in bold and italics.

**Figure 3:**
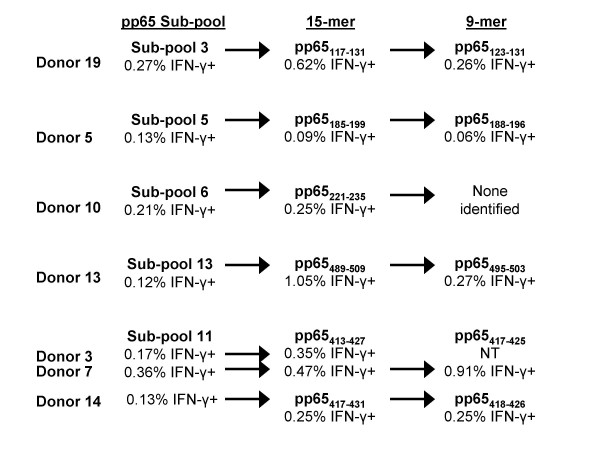
**Identification of CMV pp65 15-mer and nanomer epitopes recognized by CD8+ T cells**. PBMCs from CMV-seropositive subjects reactive with peptides in a pp65 subpool were tested against all the individual 15-mers in each subpool. Following the identification of reactive 15-mers, nanomers overlapping at 8 amino acids and spanning the reactive 15-mer peptides were tested against PBMCs from the reactive subject. The reactive 15-mer and nanomer peptides are shown. Testing of cells from 7 subjects led to the identification of 6 pp65 15-mers reactive with CD8+ T cells. Testing of the overlapping nanomers identified 5 epitopes. Cells from donor 3 were not available to test with the nanomer peptides. Although CD8+ T cells from donor 10 were reactive with peptides in subpool 6 and a reactive 15-mer, pp65_221–235_, was identified, no reactive nanomer was identified. NT = not tested.

All of the 15-mer peptides in each of the reactive subpools, 3, 5, 6, 11, and 13 were tested against PBMCs from the corresponding subjects, and we identified 6 reactive pp65 15-mer peptides (Figure 3). Four of the 6 reactive peptides each elicited a CD8+ T cell response in 1 subject, donors 5, 10, 13, and 19. The other 2 reactive peptides were overlapping 15-mers that elicited responses in donors 3, 7 and 14.

Since HLA class I antigens usually present peptides of 9 or 10 amino acids long to CD8+ T cells, nanomers were synthesized that spanned each reactive 15-mer and that overlapped by 8 amino acids. We identified 5 epitopes by testing each nanomer against PBMCs from donor(s) reactive with the corresponding 15-mer (Figure 3). Three of the 15-mers each yielded 1 epitope, pp65_123–131_, pp65_188–196_, and pp65_495–503_, in donors 19, 5, and 13, respectively (Figure 3). The nanomers spanning 2 overlapping 15-mers, pp65_413–427 _and pp65_417–431_, yielded 2 epitopes, pp65_417–425 _and pp65_418–426 _(Figure 3). However, nanomers covering pp65_221–235_, did not stimulate any cells.

Both pp65_417–425 _and pp65_418–426 _stimulated CD8+ cells from donors 3, 7, and 14, all of whom expressed HLA-B*07 (Table 6). The restriction to HLA-B*07 was confirmed by testing donor 14 PBMCs sensitized in vitro for 10 days with pp65_418–426 _against EBV-LCLs pulsed with pp65_418–426_. CD8+ IFN-γ production was detected when the in vitro sensitized PBMCs were tested against peptide-loaded EBV-LCLs expressing HLA-B*07, but not those expressing the other donor 14 HLA class I antigens. Similar epitopes, pp65_415–429 _and pp65_417–426 _have been previously found to be presented by HLA-B*07[8,19].

**Table 6:** Individual pp65 Epitopes that Induced Intracellular IFN-γ Production by CD8+ T Cells, the HLA Type of Donors With Reactive CD8+ T Cells, and HLA Antigen Restrictions of Each Epitope

**Epitope(s)**	**Donor**	HLA-A*	HLA-B*	HLA-C*	**HLA Antigen Restriction**	**Published Antigen Restriction**
pp65_123–131_	19	03, 29	**35**, 44	04, 1601	B*35	pp65_123–131 _B*3501 [20]
pp65_188–196_	05	11, 68	44, 45	05, 06	No cells available	pp65_186–196 _A*6801/2 [21]
pp65_417–425 _and pp65_418–426_	03	01, 31	**07**, 27	0202, 0702	No cells available	
	07	01, 0201	**07**, 35	04, 07	No cells available	pp65_417–426 _B*0702 [8,19]
	14	010101, 2601	**070201**, 3801	070201, 120301	HLA-B*07	
pp65 _495–503_	13	0201, 0201	15, 18	03, 07	Not tested	pp65_495–503 _A*0201 [13,30]

The HLA antigen restrictions identified are shown in bold.

The peptide pp65_123–131 _has been described as restricted to HLA-B*35, and we found this peptide to stimulate CD8+ cells from donor 19, who also expressed HLA-B*35 (Table 6)[20]. This restriction was confirmed by testing donor 19 PBMCs that were ex vivo sensitized with pp65_123–131 _against EBV-LCLs loaded with pp65_123–131. _CD8+ T cells produced IFN-γ when tested against pp65_123–131_-loaded EBV-LCLs expressing HLA-B*35 but not those expressing other donor 19 HLA class I antigens.

The epitope pp65_495–503 _stimulated CD8+ cells from donor 13, who expressed HLA-A*02 (Table 6). Since this epitope has been described by several groups as being restricted to HLA-A*02, we did not test this peptide further[8,13,21].

The epitope pp65_188–196 _stimulated CD8+ cells from donor 5, who expressed HLA-A*05; -B*11, 68, and -C*05, 06 (Table 6). Unfortunately, no PBMCs were available to test. However, pp65_186–196 _has been described as being restricted to HLA-B*35 and HLA-A*6801/02[20], so it is possible that in this donor, pp65_188–196 _is restricted to HLA-A*68[8,21].

### CD8+ T cell responses to IE-1 peptides

The 12 IE-1 subpools were tested against all 20 subjects and 4 subpools induced IFN-γ in 8 subjects (Table 7). At least 1 IE-1 subpool induced IFN-γ production in CD8+ T cells from all 8 subjects with cells reactive with the IE-1 library. One subpool stimulated CD8+ cells in 5 subjects, another stimulated CD8+ cells in 3 subjects, and 2 other subpools stimulated CD8+ cells from 1 subject (Table 7).

**Table 7:** Proportion of Peripheral Blood CD8+ T Cells From 20 Healthy CMV-Seropositive Subjects Producing Intracellular IFN-γ Following Stimulation With a Library of 120 CMV IE-1 15-mer Peptides and Each of 12 Subpools of 10 Peptides

	**CD8+ T Cells Producing Intracellular IFN-γ in Each Donor (%)**																			
	**1**	**2**	**3**	**4**	**5**	**6**	**7**	**8**	**9**	**10**	**11**	**12**	**13**	**14**	**15**	**16**	**17**	**18**	**19**	**20**

**Library**	0.05	***0.46***	***2.06***	0.04	***1.65***	***0.87***	***1.37***	0.13	0.15	***0.33***	***0.32***	0.06	0.02	0.12	***1.41***	0.05	0.05	0.02	0.03	0.08
**Subpool**																				
**1**	0.04	0.03	0.08	0.05	0.01	0.03	0.01	0.11	0.04	0.05	0.04	0.05	0.04	0.05	0.03	0.05	0.02	0.02	0.01	0.00
**2**	0.05	0.07	0.06	0.01	0.07	0.04	0.06	0.05	0.10	0.03	0.02	0.04	0.02	0.02	0.01	0.05	0.01	0.01	0.01	0.01
**3**	0.06	0.05	0.04	0.04	***0.95***	0.03	0.02	0.08	0.02	***0.26***	***0.29***	0.10	0.01	0.05	0.02	0.08	0.01	0.01	0.01	0.02
**4**	0.06	0.07	0.16	0.05	0.01	0.05	0.00	0.00	0.07	0.04	0.04	0.06	0.03	0.03	0.02	0.08	0.04	0.03	0.00	0.02
**5**	0.03	0.04	0.05	0.00	0.05	0.04	0.05	0.07	0.17	0.03	0.07	0.03	1.28	0.04	***1.60***	0.08	0.03	0.06	0.01	0.02
**6**	0.02	0.02	0.17	0.03	0.05	0.10	0.01	0.06	0.06	0.06	0.06	0.06	0.03	0.12	0.05	0.11	0.02	0.01	0.04	0.00
**7**	0.04	0.01	0.02	0.03	0.01	0.03	0.03	0.09	0.01	0.04	0.05	0.07	0.06	0.03	0.07	0.05	0.01	0.02	0.02	0.00
**8**	0.06	***0.43***	***1.65***	0.00	***0.33***	***1.05***	***1.39***	0.07	0.02	0.01	0.05	0.04	0.03	0.12	0.06	0.00	0.03	0.01	0.06	0.00
**9**	0.04	0.02	0.03	0.08	0.02	0.05	0.02	0.14	0.03	0.04	0.09	0.08	0.03	0.04	0.11	0.02	0.02	0.04	0.04	0.00
**10**	0.04	0.07	0.08	0.03	0.03	0.05	0.00	0.07	0.08	0.05	0.05	0.07	0.04	0.05	***0.25***	0.05	0.01	0.04	0.04	0.00
**11**	0.01	0.01	0.09	0.03	0.05	0.06	0.05	0.09	0.04	0.02	0.08	0.07	0.02	0.01	0.05	0.07	0.03	0.01	0.01	0.02
**12**	0.02	0.05	0.11	0.06	0.07	0.03	0.02	0.06	0.03	0.03	0.05	0.08	0.06	0.04	0.04	0.10	0.02	0.04	0.03	0.02

Positive assays are indicated in bold and italics.

**Figure 4:**
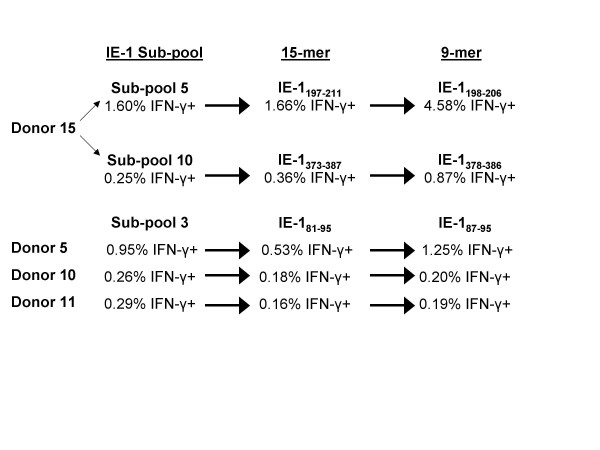
**Identification of CMV IE-1 15-mer and nanomer epitopes from 3 peptide subpools reactive with CD8+ T cells**. PBMCs from 4 donors were reactive with peptides in IE-1 subpools 3, 5, and 10. Testing of all 15-mers in each subpool identified 3 nanomers reactive with CD8+ T cells, one from each subpool. Testing of the 6 overlapping nanomers spanning each reactive 15-mer lead to the identification of 3 nanomer epitopes.

Testing the 15-mers in IE-1 subpool 5 against PBMCs from donor 15 identified IE-1_197–211 _as the reactive 15-mer and IE-1_198–206 _as the reactive nanomer (Figure 4). Testing the 15-mers in subpool 10 against cells from donor 15 revealed that IE-1_373–387 _as the reactive 15-mer and IE-1_378–386 _as the reactive nanomer (Figure 4), and testing of subpool 3 peptides against PBMCs from donors 5, 10, and 11 identified IE-1_81–95_and IE-1_87–95 _as the reactive 15-mer and nanomer (Figure 4).

**Figure 5:**
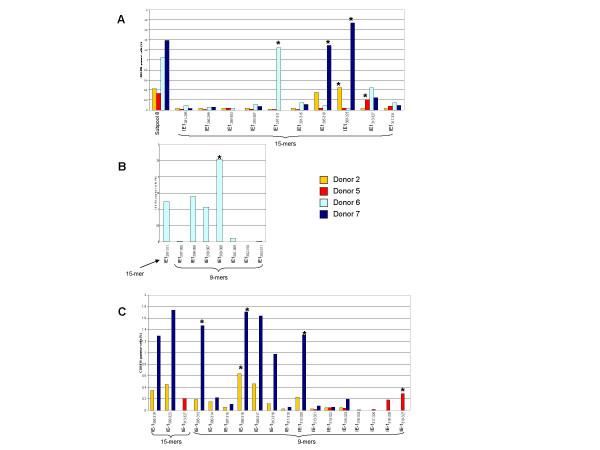
**Identification of CMV pp65 15-mer and nanomer epitopes from peptide subpool 8 that were reactive with CD8+ T cells**. Testing of IE-1 15-mers from subpool 8 against cells from donor 2 (gold bar), donor 5 (red bar), donor 6 (light blue bar), and donor 7 (navy blue bar) revealed that the peptide IE-1_297–311 _was reactive with donor 6, IE-1_305–319 _was reactive with donor 7, IE-1_309–323 _was reactive with donors 2 and 6, and IE-1_313–327 _was reactive with donor 5 (Panel A). Testing of the 6 nanomers overlapping IE-1_297–311 _against cells from donor 6 revealed that IE-1_300–308 _was the dominant nanomer (Panel B). Testing of the nanomers spanning the overlapping 15-mers IE-1_305–319_, IE-1_309–323_, and IE-1_313–327 _against PBMCs from donors 2, 5, and 7 identified 4 nanomers that were reactive with CD8+ T cells: IE-1_305–313_, IE-1_308–316_, IE-1_312–320 _and IE-1_319–327_, (Panel C).

Testing subpool 8 peptides revealed that one 15-mer and 1 nanomer were reactive with cells from donor 6, IE-1_297–311 _(Figure 5a) and IE-1_300–308 _(Figure 5b), respectively. Testing of subpool 8 peptides against cells from donors 2, 5, and 7 identified 3 reactive 15-mers: IE-1_305–319_, IE-1_309–323_, and IE-1_313–327_(Figure 5a), and 4 reactive nanomers: IE-1_305–313_, IE-1_308–316_, IE-1_312–320_, and IE-1_319–327 _(Figure 5c).

Among the 8 IE-1 epitopes identified, IE-1_87–95_, IE-1_300–308_, IE-1_305–313_, IE-1_312–320_, and IE-1_319–327 _have not been previously described. The peptide IE-1_87–95 _stimulated CD8+ cells from 3 donors, all of whom expressed HLA-C*06 (Table 8) and this restriction was confirmed by testing donor 11 in vitro sensitized PBMCs against IE-1_87–95_-loaded EBV-LCLs.

**Table 8:** Individual IE-1 Epitopes that Induced Intracellular IFN-γ Production by CD8+ T Cells, the HLA Type of Donors With Reactive CD8+ T Cells, and The HLA Antigen Restriction of Each Epitope.

**Epitope**	**Donor**	HLA-A*	HLA-B*	HLA-C*	**HLA Antigen Restriction**	**Published Antigen Restriction**
IE-1_87–95_	05	11, 68	44, 45	05, **06**		
	10	0301, 68	53, 58	04, **06**		
	11	26, 74	15, 5802	**0602**, 1601	C*0602	
IE-1_198–206_	15	0201, 30	**18, **44	0501	†	IE-1_199–207 _B*18 [22]
IE-1_300–308_	06	26, 30	35, 57	04, 18	No cells available	IE-1_297–304 _A*0201 [27]
IE-1_305–313_	07	01, 0201	07, 35	04, **07**	C*0702	
IE-1_308–316_	02	24, 32	07, 40	02, **07**	C*07	IE-1_309–317 _B*0702 [9]
	07	01, 0201	07, 35	04, **07**		
IE-1_312–320_	07	01, 0201	07, 35	04, **07**	C*07	
IE-1_319–327_	05	11, **68**	44, 45	05, 06	A*68	IE-1_316–324 _A*0201 [19]
IE-1_378–386_	15	0201, 30	**18**, 44	0501, 0501	†	IE-1_379–387 _B*18 [22]

† Cells from donor 15 did not expand when stimulated with IE-1_198–206 _and IE-1_378–386_.

The HLA antigen restrictions identified are shown in bold.

The peptide IE-1_305–313 _stimulated cells from donor 7, who expressed HLA-A*01, 0201; -B*07, 35; and -C*04, 07. Testing donor 7 PBMCs that were in vitro sensitized with IE-1_305–313 _against IE-1_305–313_-loaded EBV-LCLs confirmed that this epitope was restricted to HLA-C*07 (Table 8).

The peptide IE-1_308–316 _stimulated cells from donors 2 and 7, both expressing HLA-B*07 and -C*07. Testing donor 7 PBMCs that were in vitro sensitized with IE-1_308–316 _against IE-1_308–316_-loaded EBV-LCLs confirmed the restriction to HLA-C*07 (Table 8). However, an overlapping peptide, IE-1_309–317, _has been reported to be restricted to HLA-B*0702[9].

The peptide IE-1_312–320 _stimulated cells from donor 7 and testing of PBMCs that were in vitro sensitized with IE-1_312–320 _against peptide-loaded EBV-LCLs revealed restriction to HLA-C*07 (Table 8).

The peptide IE-1_319–327 _stimulated cells from donor 5, who expressed A*11,68; -B*44,45; and -C*05,06. Testing PBMCs that were in vitro sensitized with IE-1_319–327 _against peptide-loaded EBV-LCLs revealed that IE-1_319–327 _was restricted to HLA-A*68.

The peptide IE-1_300–308 _stimulated CD8+ cells from donor 6 however, no PBMCs were available for testing.

Among the 8 IE-1 epitopes we identified, IE-1_199–207 _and IE-1_379–386 _have been previously described as restricted to HLA-B*18 [22]. We found 2 similar epitopes reactive with donor 15 who also expressed HLA-B*18 (Table 8). Unfortunately, donor 15 PBMCs could not be expanded in vitro with either peptide, therefore we could not confirm these restrictions.

### CD4+ T cell responses to pp65 peptides

After testing pp65 peptide subpools against PBMCs from all 20 seropositive subjects, 5 subpools stimulated CD4+ cells in each of the 10 subjects who were responsive to the pp65 library (Table 9). Subpool 8 stimulated CD4+ cells from 1 subject, subpool 6 from 3 subjects, subpool 10 from 4 subjects, and subpool 13 from 5 subjects (Table 9).

**Table 9:** Proportion of Peripheral Blood CD4+ T Cells From 20 Healthy CMV-Seropositive Subjects Producing Intracellular IFN-γ Following Stimulation With a Library of 138 CMV pp65 15-mer Peptides and Each of 14 subpools of 10 Peptides*

	**CD4+ T Cells Producing Intracellular IFN-γ in Each Donor (%)**																			
	**1**	**2**	**3**	**4**	**5**	**6**	**7**	**8**	**9**	**10**	**11**	**12**	**13**	**14**	**15**	**16**	**17**	**18**	**19**	**20**

**Library**	***0.16***	0.06	0.00	0.06	0.11	***0.76***	***0.11***	***0.65***	0.08	***0.21***	***3.33***	***0.18***	***0.20***	***1.34***	0.23	0.02	***0.15***	0.03	0.04	0.01
**Subpool**																				
**1**	0.01	0.01	0.02	0.01	0.02	0.03	0.00	0.03	0.02	0.01	0.03	0.05	0.08	0.05	0.04	0.00	0.02	0.01	0.01	0.04
**2**	0.05	0.09	0.01	0.03	0.05	0.05	0.02	0.04	0.02	0.01	0.03	0.06	0.02	0.06	0.03	0.02	0.03	0.04	0.01	0.03
**3**	0.03	0.02	0.01	0.01	0.03	0.03	0.04	0.05	0.05	0.03	0.03	0.06	0.03	0.06	0.06	0.00	0.03	0.02	0.04	0.02
**4**	0.01	0.06	0.02	0.01	0.03	0.06	0.00	0.09	0.01	0.02	0.04	0.04	0.01	0.03	0.04	0.01	0.01	0.02	0.02	0.00
**5**	0.02	0.04	0.01	0.01	0.02	0.02	0.00	0.04	0.04	0.01	0.04	0.05	0.02	0.07	0.02	0.02	0.01	0.00	0.01	0.01
**6**	0.02	0.04	0.01	0.04	0.01	0.03	0.01	***0.45***	0.04	***0.11***	0.02	***0.18***	0.02	0.01	0.02	0.02	0.01	0.04	0.01	0.03
**7**	0.01	0.05	0.04	0.01	0.01	0.02	0.02	0.03	0.01	0.07	0.02	0.06	0.05	0.04	0.07	0.02	0.02	0.07	0.02	0.01
**8**	0.04	0.04	0.01	0.03	0.02	0.09	***0.05***	0.12	0.01	0.01	0.04	0.08	0.00	0.07	0.05	0.02	0.01	0.03	0.01	0.03
**9**	0.06	0.13	0.03	0.03	0.03	0.05	***0.05***	0.04	0.03	0.01	0.05	0.05	0.01	0.06	0.02	0.02	0.01	0.03	0.02	0.09
**10**	***0.14***	0.02	0.00	0.03	0.05	***0.56***	0.00	0.10	0.05	0.04	***2.76***	0.08	0.01	***0.96***	0.03	0.02	0.02	0.01	0.02	0.00
**11**	0.03	0.02	0.00	0.00	0.02	0.15	0.00	0.08	0.01	0.01	0.06	0.13	0.01	0.07	0.01	0.01	0.01	0.01	0.00	0.01
**12**	0.04	0.03	0.01	0.00	0.02	0.04	0.01	0.05	0.04	0.01	0.01	0.04	0.00	0.06	0.03	0.02	0.02	0.01	0.01	0.05
**13**	0.02	0.03	0.02	0.03	0.03	***0.27***	0.00	0.13	0.07	0.07	0.05	***0.12***	***0.14***	***0.42***	0.13	0.01	***0.12***	0.02	0.01	0.00
**14**	0.03	0.06	0.02	0.01	0.02	0.09	0.00	0.11	0.07	0.01	0.03	0.08	0.01	0.09	0.02	0.01	0.01	0.01	0.02	0.00

* Subpool 14 contained 8 peptides

Positive assays are indicated in bold and italics.

**Figure 6:**
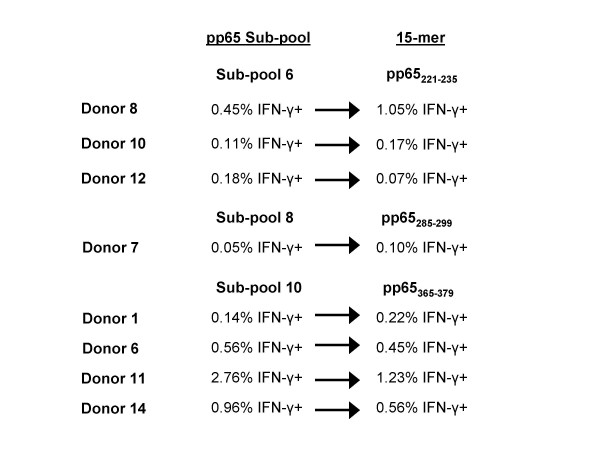
**Identification of CMV pp65 15-mer epitopes from 3 peptide subpools that were reactive with CD4+ T cells**. PBMCs from 8 donors were reactive with peptides in subpools 6, 8, and 10. Testing of all 15-mers in each subpool identified three15-mers that were reactive with CD4+ T cells, one from each subpool.

**Figure 7:**
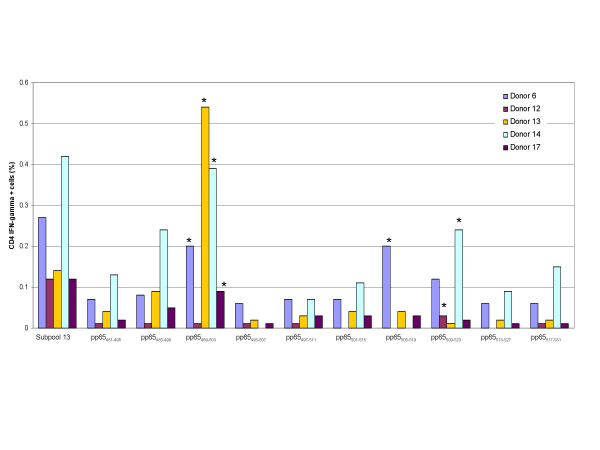
**Identification of CMV pp65 15-mer epitopes from peptide subpool 13 that were reactive with CD4+ T cells**. PBMCs from 5 donors were reactive with pp65 peptides in subpool 13. Testing of all 15-mers in subpool 13 identified three 15-mers that were reactive with CD4+ T cells, pp65_489–503_, pp65_505–519_, and pp65_509–523_.

All 15-mers in each reactive subpool were tested against PBMCs from the 10 subjects, revealing a total of 6 reactive pp65 15-mer epitopes. The peptide pp65_221–235 _was identified as the reactive 15-mer in subpool 6, pp65_285–299 _in subpool 8, and pp65_365–379 _in subpool 10 (Figure 6). Subpool 13 yielded 3 reactive 15-mers; pp65_489–503_, pp65_505–519_, and pp65_509–523 _(Figure 7).

Epitope pp65_221–235 _reacted with PBMCs from donors 8, 10, and 12 (Table 10). Donor 12 PBMCs were in vitro sensitized with pp65_221–235_, and reacted with pp65_221–235_,-loaded EBV-LCLs expressing DRB1*15 and DRB5*01. It is likely that pp65_221–235 _is presented by DRB1*15 since 2 of the 3 donors expressed DRB1*15 but only donor 12 expressed DRB5*01. In addition, pp65_225–239 _has previously been reported to be restricted to DRB1*15[23].

**Table 10:** Individual pp65 Epitopes that Induced intracellular IFN-γ Production by CD4+ T Cells, the HLA Type of Donors With Reactive CD4+ T Cells, and the HLA Antigen Restriction of Each Epitope

**Peptide(s)**	**Donor**	DRB1*	**DRB-**	DQB1*	**HLA Antigen Restriction**	**Published Antigen Restriction**
	12	03021, **1503**	3*01, 5*01	06	DRB1*15 or DRB5*01	pp65_225–239 _DR15 [23]
pp65_221–235_	08	13031, **1503**	3*02, 3*02	02, 06		
	10	0701, 1302	3*0301, 4*0401	0202, 0604	No cells available	
pp65_285–299_	07	**0401, 0701**	4*01, 4*01	0303, 03	No cells available	pp65_281–295 _DR4, 7 [11]
pp65_365–379_	01	0901, **11**	3*00, 4*01	02, 06		pp65_361–376 _DR11 [24]
	06	**11**, 16	3*02, 5*02	0301, 05		
	11	0102, **1101**	3*0202	0301, 0501	DRB1*11	pp65_361–375 _DR11 [11]
	14	0402, **1101**	3*02, 4*0103	0301, 0302		
pp65_489–503_	06	11, 16	3*02, 5*02	0301, 05	DRB3*02	
	13	13, 1501	3*02, 5*01	06		pp65_489–503_
	14	0402, 1101	3*02, 4*0103	0301, 0302		DR11,3 [24]
	17	13,1503	3*02, 5*01	06		pp65_505–523 _DR52 [11]
pp65_505–519_	06	11, 16	3*02, 5*02	0301, **05**	DQB1*0502	pp65_505–523 _DR1 [23]
pp65_509–523_	12	03021, 1503	3*01, 5*01	06	DRB3*0101	
	14	0402, 1101	3*02, 4*0103	0301, 0302	No cells available	pp65_509–524 _DR3 [24]

The HLA antigen restrictions identified are shown in bold

Pp65_365–379 _stimulated cells from 4 donors all expressing HLA-DRB1*11 (Table 10). PBMCs from donor 11 were in vitro sensitized with pp65_365–379_, and produced IFN-γ when tested against pp65_365–379 _-loaded EBV-LCLs expressing HLA-DRB1*11, but not when tested against those expressing HLA-DRB1*0102, DRB3*0202, DQB1*0301, and DQB1*0501. The testing of this peptide against a second subject, donor 6, revealed similar results. Two similar epitopes, pp65_361–376 _and pp65_361–375_, have been described as restricted to HLA-DR11[11,24].

Epitope pp65_489–503 _reacted with 3 donors, all of whom expressed HLA-DRB3*02 (Table 10). Donor 6 PBMCs that were in vitro sensitized with pp65_489–503_, produced IFN-γ when tested against EBV-LCLs expressing HLA-DRB3*02, but not when tested against the other donor 6 class II antigens. Although we found that pp65_489–503 _was restricted to DRB3*02, others have described this peptide as restricted to DRB1*11 and DRB1*03 [24].

Epitope pp65_505–519 _was reactive with PBMCs from donors 6 and 17 and testing of donor 6 pp65_505–519 _ex vivo sensitized PBMCs against pp65_505–519_-loaded EBV-LCLs revealed presentation by DQB1*0502. Donor 17 did not express DQB1*0502, but cells were not available for additional testing. The epitope pp65_505–523 _has been reported to be restricted to DRB1*01[23] and DR52[11].

Epitope pp65_509–523 _reacted with PBMCs from donors 12 and 14, and testing donor 12 in vitro sensitized PBMCs against loaded EBV-LCLs revealed presentation by DRB3*0101. Donor 14 did not express DRB3*0101 however, an adequate number of cells were not available for in vitro sensitization and testing. The epitope pp65_509–524 _was previously reported to be restricted to DRB1*03[24].

Pp65_285–299 _reacted with cells from donor 7, who expressed both HLA-DRB1*0401 and HLA-DRB1*0701 (Table 10). Although additional PBMCs were not available for testing, a similar epitope, pp65_281–295_, was previously found to be restricted to both HLA-DR*4 and HLA-DR*7[11].

### CD4+ T cell responses to IE-1

Testing with both the IE-1 library and the 12 subpools did not reveal active CD4+ populations from any subjects (Table 11).

**Table 11:** Proportion of Peripheral Blood CD4+ T Cells From 20 Healthy CMV-Seropositive Subjects Producing Intracellular IFN-γ Following Stimulation With a Library of 120 CMV IE-1 15-mer Peptides 15 and Each of 12 Subpools Made Up of 10 Peptides

	**CD4+ T Cells Producing Intracellular IFN-γ in Each Donor (%)**																			
	**1**	**2**	**3**	**4**	**5**	**6**	**7**	**8**	**9**	**10**	**11**	**12**	**13**	**14**	**15**	**16**	**17**	**18**	**19**	**20**

**Library**	0.02	0.04	0.01	0.03	0.02	0.14	0.03	0.05	0.01	0.02	0.08	0.06	0.04	0.13	0.09	0.02	0.05	0.02	0.00	0.10
**Subpool**																				
**1**	0.02	0.06	0.05	0.01	0.01	0.02	0.01	0.11	0.02	0.01	0.04	0.02	0.01	0.04	0.03	0.01	0.01	0.02	0.00	0.00
**2**	0.02	0.01	0.05	0.02	0.01	0.03	0.01	0.00	0.02	0.01	0.02	0.03	0.01	0.02	0.04	0.01	0.01	0.02	0.00	0.01
**3**	0.04	0.00	0.13	0.02	0.01	0.01	0.00	0.10	0.02	0.02	0.03	0.05	0.05	0.02	0.04	0.02	0.03	0.01	0.01	0.00
**4**	0.06	0.05	0.01	0.04	0.01	0.06	0.03	0.05	0.04	0.01	0.03	0.04	0.01	0.05	0.03	0.02	0.02	0.02	0.00	0.03
**5**	0.02	0.06	0.04	0.01	0.02	0.04	0.02	0.06	0.07	0.01	0.04	0.03	0.02	0.05	0.04	0.01	0.02	0.02	0.00	0.03
**6**	0.03	0.00	0.04	0.01	0.02	0.15	0.01	0.04	0.00	0.02	0.05	0.04	0.03	0.08	0.03	0.02	0.01	0.02	0.02	0.01
**7**	0.04	0.02	0.02	0.01	0.01	0.03	0.00	0.09	0.01	0.01	0.04	0.03	0.02	0.07	0.04	0.04	0.02	0.01	0.01	0.00
**8**	0.03	0.03	0.00	0.01	0.00	0.02	0.01	0.03	0.03	0.01	0.05	0.01	0.01	0.03	0.03	0.01	0.00	0.00	0.01	0.03
**9**	0.03	0.03	0.01	0.01	0.02	0.04	0.00	0.07	0.01	0.01	0.07	0.07	0.02	0.03	0.09	0.01	0.01	0.00	0.02	0.01
**10**	0.03	0.05	0.01	0.00	0.02	0.02	0.02	0.06	0.02	0.01	0.04	0.03	0.01	0.05	0.02	0.02	0.02	0.01	0.01	0.01
**11**	0.01	0.04	0.00	0.00	0.01	0.04	0.01	0.07	0.01	0.02	0.04	0.03	0.00	0.02	0.02	0.01	0.00	0.01	0.01	0.01
**12**	0.01	0.03	0.01	0.04	0.02	0.02	0.00	0.10	0.03	0.01	0.04	0.04	0.02	0.01	0.04	0.00	0.02	0.01	0.00	0.01

## Discussion

The aim of this investigation was to map CMV pp65 and IE-1 epitopes in 20 CMV-seropositive healthy subjects in order to identify peptides that may be important in vaccination, adoptive immunotherapy, and the monitoring of transplant recipients. Among the 20 subjects, CD8+ T cell responses to pp65 and IE-1 were detected in a similar proportion of patients: 35% versus 40%. However, CD4+ T cell responses to pp65 were more common than responses to IE-1, as 50% of subjects reacted to pp65 but none reacted to IE-1. Using similar methodology, Kern et al also found CD8+ T cell responses in healthy subjects to both pp65 and IE-1[9,11]. While they did not specifically screen for IE-1 epitopes that were presented by CD4+ T cells, a comparison of CD4+ T cell responses to pp65 peptides with responses to CMV lysate suggested that pp65 was a dominant target of the CMV-specific CD4+ T cell response[11]. A recent analysis of overlapping 15-mers spanning 213 CMV open reading frames including pp65 and IE-1, by Sylwester et al found that the CD8+ and CD4+ T cell responses were directed to peptides in a variety of proteins,[25]. In addition, they found that CD8+ T cell responses to both pp65 and IE-1 were strong and the CD4+ T cell responses were strong to pp65 but weak to IE-1 which supports the results of our study [25].

We identified 4 pp65 and 8 IE-1 nanomers presented by HLA class I, and 6 pp65 15-mers presented by HLA class II. While all 4 of the pp65 CMV class I epitopes have been previously described, 6 of the 8 IE-1 class I epitopes, and 2 of the 6 pp65 class II epitopes have not. The pp65 epitopes and restrictions that we identified were similar or identical to those reported previously, but the IE-1 class I epitopes had either not been described or we identified HLA restrictions that differed from those reported.

Four of the 6 new IE-1 class I epitopes we identified were restricted to HLA-C antigens, a finding that may be correlated to the fact that all of our subjects and EBV-LCLs were HLA typed at high-resolution. Because HLA-C antigen typing results were ambiguous prior to the advent of molecular genotyping, the availability of precise molecular typing for this study may have permitted the identification of epitopes not possible in the past when molecular methods were not available or were less precise.

While the conventional thought has been that unique peptides are each presented by a specific HLA allele, some CMV epitopes are presented by multiple alleles. Epitopes for HLA class I antigens are generally believed to be very restrictive, with epitopes binding to specific HLA-types. Our results suggest that this is not entirely true for IE-1 class I epitopes as the HLA restrictions were somewhat promiscuous. We found that the epitope IE-1_319–327 _was restricted to HLA-A*68, while a similar epitope IE-1_316–324 _has been described as restricted to A*0201. We also found that IE-1_308–316 _was restricted to HLA-C*07, but IE-1_309–317 _is reported to be restricted to HLA-B*0702 [19]. In previous studies, we found that the epitopes pp65_341–349 _and pp65_341–350 _are presented by HLA-A*0101, HLA-A*2402, and HLA-Cw*0402 [14,26].

We found that 1 region of IE-1, amino acid residues 300 to 327, to be more immunogenic than other regions. We identified 5 epitopes in this region, in addition to the 3 other epitopes previously identified[9,19,27]. This region was particularly immunogenic for HLA-C*07 as we identified 3 HLA-C*07 restricted epitopes in this region.

Although pp65_495–503 _has been found to presented by HLA-A*02[8,13], the 15-mer in our pp65 library that encompassed this epitope, pp65_489–503_, stimulated CD8+ T cells from only 1 of 6 HLA-A*02 CMV seropositive subjects tested. To determine if the lack of response to pp65_489–503 _was because the cells were not responsive to pp65_495–503 _or because the epitope was not presented in the context of the 15-mer, we tested the 9-mer, pp65_495–503_, against PBMCs for all 5 HLA-A*02 subjects who had not responded to pp65_489–503_. Similarly, CD8+ cells from none of these subjects produced significant quantities of intracellular IFN-γ in response to pp65_495–503 _stimulation. Our study differed from most other studies in that we screened the peptide libraries against cells that had not been sensitized in vitro, while the studies that first identified pp65_495–503 _as an immune dominant HLA-A*02 restricted epitope used T cells that had been stimulated with CMV-infected fibroblasts[8,13]. These results suggest that the baseline frequency of CTLs in HLA-A*0201 healthy subjects that are restricted to pp65_495–503 _is usually low, but expand robustly when challenged with CMV antigens.

To provide optimum protection from CMV infection and disease, vaccines that elicit both CD8+ and CD4+ responses are required[5,23]. Cytotoxic T cells (CTL) prevent CMV infection from developing into CMV disease, and CD4+ T cell responses maintain CTLs[5]. Several vaccines to CMV have been tested and found to have some efficacy, including live attenuated vaccines, subunit vaccines, and peptide vaccines[28]. Subunit vaccines have focused on gB glycoprotein because it is the primary target of natural antibodies, and pp65 because it is the major target of CTLs. Additionally, pox virus-vectored vaccines expressing pp65 have stimulated CD8+ T cell responses in CMV-seronegative subjects. Our finding of naturally occurring CD8+ and CD4+ T cell responses to pp65 in healthy subjects also supports its use in subunit vaccines. The naturally occurring CD8+ T cell responses to IE-1 that we found in a large proportion of healthy subjects supports IE-1 addition to vaccines. In fact, a trivalent DNA vaccine containing gB, pp65, and IE-1 is being tested in phase I clinical trials[28].

In one study of lung and heart transplant recipients have revealed that protection from CMV disease is more closely associated with the CD8+ T cell responses to IE-1 than to pp65, indicating that IE-1 is a good candidate for CMV vaccines[29]. However, the lack of naturally occurring CD4+ T cell responses to IE-1 suggests that it may not be as useful as pp65 in a monovalent vaccine since both CD8+ and CD4+ responses to pp65 are frequently found in healthy CMV-seropositive positive subjects.

The characterization of pp65 and IE-1 epitopes will be valuable for monitoring patients treated with vaccines or adoptive immunotherapies, and these epitopes alone or in conjunction with HLA class I tetramers or pentamers can be used to follow the immune responses to these therapies. In addition, peptides, and HLA tetramers and pentamers can be used to assess the host immune response to CMV.

In conclusion, by screening healthy, CMV-seropositive subjects with pp65 and IE-1 peptides, we identified several new IE-1 epitopes and confirmed several other IE-1 and pp65 epitopes. Similar numbers of CMV pp65 and IE-1 HLA class I epitopes were detected, and one region of IE-1 was rich in HLA class I epitopes, especially those restricted to HLA-C*07. Although pp65 was rich in HLA class II epitopes, no IE-1 class II epitopes were identified.

## Competing interests

The author(s) declare that they have no competing interests.

## Authors' contributions

MB, FMM and DFS conceived of the study; SLS and MB performed the research and collected data; SLS, MB, SS, FMM and DFS analyzed data; SA provided vital new reagents and participated in study design; SLS, DFS and MB drafted the manuscript; and all authors checked the final version of the manuscript.
